# Rapid construction of mycobacterial mutagenesis vectors using ligation-independent cloning

**DOI:** 10.1016/j.mimet.2010.07.014

**Published:** 2010-10

**Authors:** Ricardo Balhana, Neil G. Stoker, Mahmudul Hasan Sikder, Francois-Xavier Chauviac, Sharon L. Kendall

**Affiliations:** a Department of Pathology and Infectious Diseases, The Royal Veterinary College, Centre for Emerging, Endemic and Exotic Disease, Hawkshead Lane, Hertfordshire, AL9 7TA, United Kingdom; b School of Crystallography, Birkbeck College, Malet Street, London WC1E 7HX, United Kingdom

**Keywords:** Mycobacteria, Homologous recombination, Ligation independent cloning, Gene deletion

## Abstract

Targeted mutagenesis is one of the major tools for determining the function of a given gene and its involvement in bacterial pathogenesis. In mycobacteria, gene deletion is often accomplished by using allelic exchange techniques that commonly utilise a suicide delivery vector. We have adapted a widely-used suicide delivery vector (p1NIL) for cloning two flanking regions of a gene using ligation independent cloning (LIC). The pNILRB plasmid series produced allow a faster, more efficient and less laborious cloning procedure. In this paper we describe the making of pNILRB5, a modified version of p1NIL that contains two pairs of LIC sites flanking either a *sacB* or a *lacZ* gene. We demonstrate the success of this technique by generating 3 mycobacterial mutant strains. These vectors will contribute to more high-throughput methods of mutagenesis.

## 1 Introduction

The repertoire of molecular tools available for the study of mycobacteria is narrow when compared to those available for other bacterial species such as *Escherichia coli* and *Bacillus subtilis*. Such tools are needed to investigate the many mycobacterial species involved in human disease. The construction of targeted mycobacterial mutants has been invaluable for the understanding of gene function, the demonstration of gene essentiality and in the generation of attenuated strains for vaccine trials (Machowski et al., 2005). Methods for targeted mutagenesis in mycobacteria use allelic exchange via suicide, or conditionally replicating, plasmid vectors or phages (Bardarov et al., 2002; Parish and Stoker, 2000; Pelicic et al., 1997, Sander et al., 1995). The method used in our laboratory is a two-step mutagenesis procedure that allows efficient production of unmarked mutants through (1) integration of the mutagenesis plasmid in the target gene by a single cross-over (SCO), and (2) isolation of double cross-overs (DCOs) through negative selection (Parish et al., 1999, Parish and Stoker, 2000). This system has been used widely for the generation of unmarked mycobacterial mutants (Boshoff et al., 2003, Chauhan et al., 2006, Curry et al., 2005, Dawes et al., 2003, Hu et al., 2006, Matsoso et al., 2005; Onwueme et al., 2004, Parish et al., 2005; Parish et al., 2003, Primm et al., 2000, Shimono et al., 2003, Smith et al., 2001).

Although the mutagenesis procedure is quite efficient, the construction of the mutagenesis vector can be difficult. The target gene, and flanking regions, are cloned into a manipulation plasmid (pNIL series) and mutated, or two flanking regions are cloned in separately without the gene. This latter approach is potentially the most useful, but cloning relies on two directional cloning procedures, and in our experience this is not always easy, both because it can be technically difficult, and because it may be constrained by restriction sites in the sequences to be cloned.

**Fig. 1:**
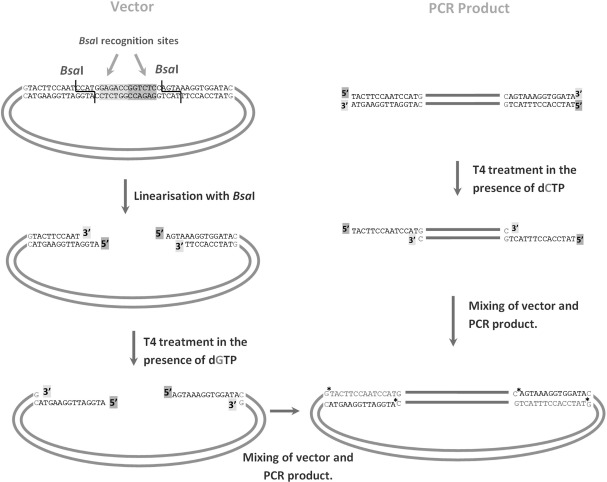
Illustration of how LIC works using the enzyme *Bsa*I for the linearisation of the vector. The plasmid contains two *Bsa*I sites that once digested will expose 3′ ends accessible to T_4_ DNA polymerase. If only dGTP is supplied, the polymerase will digest each strand from the 3′ terminus until it finds a guanine residue. The process originates a long overhang on the 5′ ends of the vector. The same approach is used for the PCR products, which are also treated with T_4_ DNA polymerase, in the presence of dCTP this time. The 5′ overhangs generated are complementary to those obtained in the vector and therefore the structure will anneal together. (*SS break, this breaks are repaired in vivo after transformation).

Recently, ligation-independent cloning (LIC) methods have began to supersede conventional restriction digestion/ligation approaches and provide a more reliable high-throughput approach (Dan et al., 2009, Eschenfeldt et al., 2009, Lee and Kim, 2009, Qin et al., 2008). LIC relies on the generation of long, compatible, cohesive ends between a vector and a PCR insert. (Aslanidis and de Jong, 1990, Haun et al., 1992, Yang et al., 1993). A type IIS restriction enzyme (such as *Bse*RI) is normally used; these enzymes cut outside their recognition sequences, and long single strands (usually about 12 nucleotides long) can be produced at each end through the exonuclease activity of T_4_ DNA polymerase in the presence of a sole nucleotide. Complementary single strands are generated in a PCR product by designing appropriate primers, and the two molecules are annealed. Due to the large number of hydrogen bonds formed between the termini of the linearised vector and target insert, the molecules anneal together sufficiently strongly for subsequent procedures without the requirement for covalent joining by DNA ligase. The structure can be transformed efficiently and subsequently repaired within the cell. This approach eliminates the requirement for restriction enzyme-generated cohesive ends and a separate ligation step. An illustration of this process is given in Fig. 1.

Here we describe adapted pNIL vectors that allow simple ligation-independent cloning of two flanking regions of DNA, making this part of the mutagenesis trivial in most cases. We demonstrate the successful use of these vectors in the construction of 3 mutants of *Mycobacterium smegmatis*. These vectors will contribute to more high-throughput methods of mutagenesis and we hope will be an invaluable tool for the mycobacterial research community.

## 2 Methods

### 2.1 Bacterial strains and culture conditions

The strains and plasmids used in this study are described in Table 1. All bacterial cultures were grown at 37 °C and liquid cultures were grown with shaking (250 rpm). *E. coli* DH5α was used as a strain for cloning. *E. coli* strains were grown in a Luria–Bertani medium. *M. smegmatis* mc^2^155 was grown in Middlebrook 7H9 broth (Difco) containing 10% oleic acid-albumin-dextrose-catalase supplement (OADC) (Becton Dickinson) and 0.05% Tween 80 or Middlebrook 7H11 agar containing 10% OADC. Hygromycin (50 μg/ml) kanamycin (50 μg/ml for *E. coli* and 20 μg/ml for *M. smegmatis*), 5-bromo-4-chloro-3-indolyl-β-D-galactopyranoside (Xgal, 50 μg/ml) and sucrose (5% w/v for *E. coli* and 2% w/v for *M. smegmatis* ) were used for selection as appropriate.

### 2.2 Oligonucleotides used in this study

A list of oligonucleotides and their use in this study is given in Tables 2 and 3.

### 2.3 Introduction of ligation-independent cloning (LIC) sites into p1NIL

**Fig. 2:**
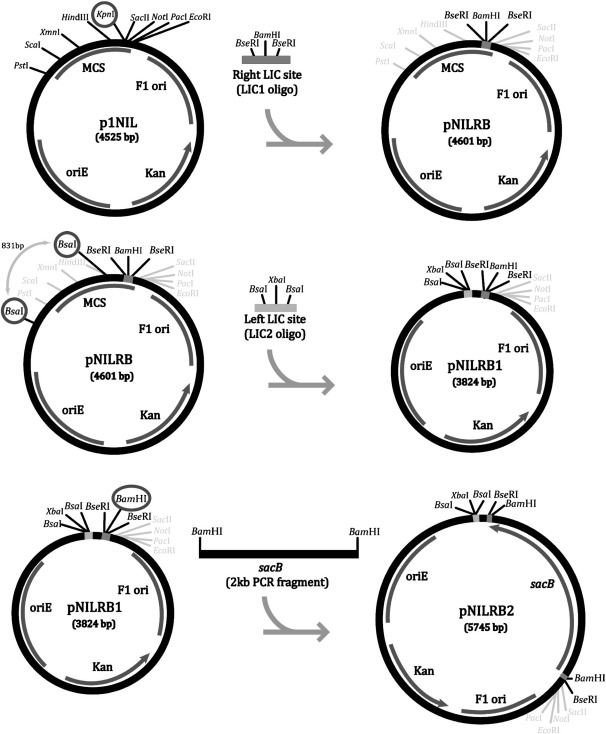
Diagram illustrating the cloning steps necessary to create pNILRB2. The right and left LIC site oligonucleotides were synthesized with overhangs compatible with those generated by the enzymes used to cut the plasmid. A *sacB* PCR product was then inserted in the *Bam*HI site of the right LIC site to construct pNILRB2.

**Fig. 3:**
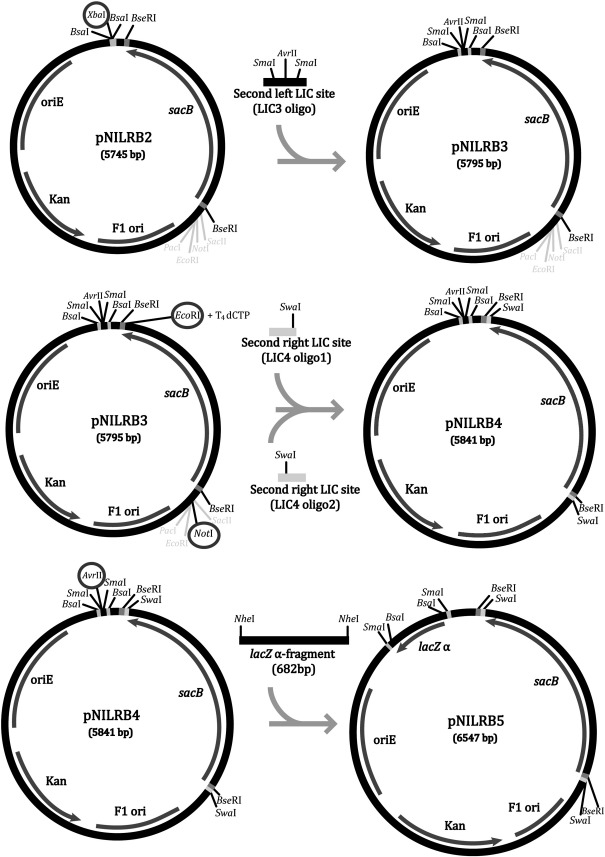
Diagram illustrating the cloning steps necessary to create pNILRB5. One extra left LIC oligonucleotide with compatible overhangs was added to pNILRB2 once the later is digested with *Xba*I, therefore creating pNILRB3. Subsequently, 2 other LIC oligonucleotides were added adjacent to the *sacB* gene creating a fourth LIC site, the resulting plasmid, pNILRB4. Finally a *lac*Z PCR fragment was added between the two left LIC sites using the enzyme *Avr*II. This last step produced pNILRB5.

DNA manipulation techniques were performed as described (Sambrook and Russell, 2001). The cloning strategy to build the LIC vectors is illustrated in Figs. 2 and 3. The pNILRB vectors were obtained using p1NIL (Parish and Stoker, 2000) as a backbone. Because individual gene flanks might be cloned, initially the vector was adapted to contain two LIC sites. The first LIC site (LIC1) was introduced by digesting p1NIL with *Kpn*I and cloning in phosphorylated and annealed synthetic oligonucleotides described in Table 2. This resulted in vector pNILRB. In order to introduce a second LIC site, a second set of LIC oligonucleotides (LIC2) was phosphorylated, annealed and introduced into the *Bsa*I digested pNILRB. This resulted in the conconmitant removal of an 831 bp fragment and the generation of pNILRB1 possessing two LIC sites for gene flank cloning (Note, introduction of the LIC sites deleted most of the multiple cloning site of p1NIL, Fig. 2).

Although LIC cloning is efficient due to the fact that the digested vector will not reanneal, it is useful to have a selective marker that can be used to select for the presence of the insert. A *sacB* gene was amplified from pNIC28-BSA4 (GenBank accession no. EF198106) using the primers listed in Table 3. The PCR product was cut with *Bam*HI and ligated to *Bam*HI-digested pNILRB1. This resulted in the insertion of the marker into LIC2 site and generating pNILRB2.

To increase the flexibility of the vector and facilitate DNA manipulations, two extra LIC sites were added for the cloning of each flank. pNILRB2 was digested with *Xba*I to insert an alternative LIC site for cloning of upstream gene flanks, using the synthetic phophorylated/annealed oligonucleotides listed in Table 2 (LIC3). The resulting vector, termed pNILRB3 contains a total of 3 LIC sites. The final LIC site (LIC4) was split-cloned in either side of the *sacB* gene. In order to add the final site, pNILRB3 was digested with *Eco*RV (at the end of the *sacB* gene) and treated with T_4_ DNA polymerase for 30 min at 22 °C in the presence of dCTP to originate an overhang compatible with oligonucleotides containing half of LIC4 (SwaI_LICR/SwaI_LICR_COMP, Table 2). The vector resulting from this insertion was cut with *Not*I (before the start site of the *sacB* gene) for the insertion of the second half of the LIC site using the phosphorylated and annealed oligonucleotides (SwaI_LICL/SwaI_LICL_COMP, Table 2). The resulting construct *(*pNILRB4)*,* contained 2 LIC sites available per flank and the possibility of negative selection with sucrose for the inclusion of right gene flanks.

Finally, in order to introduce a selective marker for the cloning of the left flanks, an α-fragment of a *lacZ* gene was introduced into pNILRB4. The *lacZ* α-fragment was PCR amplified from the plasmid pUC18 (Yanisch-Perron et al., 1985) using the pair of primers listed in Table 3. The PCR product was digested with *Nhe*I to produce compatible overhangs with *Avr*II-digested pNILRB4. The two fragments were ligated originating pNILRB5, which possesses four possible LIC sites and selective markers for cloning of both, upstream and downstream gene flanks.

### 2.4 Linearisation of vectors for LIC cloning

LIC vectors were digested either with *Bsa*I or *Bse*RI to expose their 3′ ends which were treated with T_4_ DNA polymerase in an 80 μl total volume. Briefly, 2–3 μg of hydrolyzed DNA were incubated for 30 min at 22 °C with 12 U of T_4_ DNA polymerase (New England Biolabs) in the presence of the provided buffer and additional 5 mM dGTP, 10 mM DTT and 15 μM BSA. The reaction was stopped by heat inactivation at 75 °C for 20 min. This generated a LIC-ready vector with 5′ overhangs of 14 nucleotides.

### 2.5 PCR products for LIC cloning

PCR amplification of gene flanks was carried out with primers where a gene specific sequence of 18–20 nucleotides was extended on the 3′ end with 5′TACTTCCAATCCATG3′ for the sense primer and 5′TATCCACCTTTACTG3′ for the antisense primer, when cloning upstream gene flanks (using the LIC site containing *Bsa*I), and with 5′TATCCACCTTTACTG3′ for the sense primer along with 5′TACTTCCAATCCATG3′ for the antisense primer, when cloning downstream flanks (using the LIC site of the vector that contains *Bse*RI). Accordingly, primer pairs for gene flanks that were selected to test the LIC vectors are listed in Table 3.

The PCR products obtained were treated with T_4_ DNA polymerase similarly to the vectors but using dCTP as the sole nucleotide in a 20 μl volume. Briefly, 3 μg of DNA was incubated for 30 min at 22 °C with 9 U of T_4_ DNA polymerase (New England Biolabs) in the presence of the provided buffer and additional 5 mM dGTP, 10 mM DTT, 15 μM BSA. The reaction was stopped by heat inactivation at 75 °C for 20 min.

### 2.6 Ligation-independent cloning of target gene flanks

Linearised vector and PCR products resulting from T_4_ DNA polymerase treatment were mixed together in a molarity proportion of 1:10 (vector:insert) and annealed at room temperature for 10 min, followed by an 8 min incubation on ice. The annealing mix was directly transformed (Sambrook and Russell, 2001) into DH5α *E. coli* competent cells (Invitrogen). The two flanks of each gene were cloned sequentially, having one transformation between each cloning step.

### 2.7 Construction of knock-out mycobacterial strains using LIC delivery vectors

LIC constructs containing both flanks of a gene (without the gene itself) were hydrolyzed with *Pac*I and ligated with a marker cassette originated from the digestion of pGOAL19 with *Pac*I as described (Parish and Stoker, 2000). The final suicide constructs, containing the appropriate selection genes, were used to electroporate *M. smegmatis* mc^2^ 155 and mutants were isolated as described (Parish and Stoker, 2000). Briefly, UV treated DNA (100 mJ cm^−2^) or non-UV treated DNA was electroporated (Parish et al., 1999) and hygromycin/kanamycin/*lacZ* transformants were re-streaked to allow a second cross-over event to occur. A loopful of cells was re-suspended in a liquid medium and thoroughly vortexed with 1/4 ml of 1 mm glass beads. Serial dilutions were plated onto sucrose/X-Gal and white colonies were selected and tested for kanamycin sensitivity.

Strains were genotyped using PCR with genomic DNA template and the products with the correct size containing flanks and deletion region were sequenced.

## 3 Results

### 3.1 Construction of LIC vectors for generation of gene deletions in mycobacteria

To create a LIC vector, we aimed to insert two different LIC sites into our starting vector, p1NIL (Fig. 2). This would allow separate cloning of upstream and downstream regions of the gene to be mutated. p1NIL was firstly adapted to introduce one LIC site into the unique *Kpn*I site, based on the LIC sequences used previously in the LIC expression vector pET21a-LIC (GenBank accession EF456737, Fig. 2). This region now contained two *Bse*RI (recognition site GAGGAG) sequences for the linearisation of the vector flanking sequences (Table 2) for T_4_ polymerase-driven overhang generation. The resulting plasmid was named pNILRB.

A different type IIS restriction enzyme (*Bsa*I; recognition site GGTCTC) was used as the basis for the second LIC site. It happens that the plasmid already had two *Bsa*I sites in the required orientation near the multiple cloning site, and these were replaced with a synthetic fragment, based on the LIC sequences used previously in the LIC expression vector pNIC28-BSA4 (GenBank accession no. EF198106, Fig. 2). The second LIC site was introduced by replacement of the 831 bp sequence in the vector flanked by these native *Bsa*I sites, and is located 74 bp from the first LIC site. The resulting construct, pNILRB1, is a fully functional LIC mutagenesis vector where one gene flank can be cloned using the *Bsa*I-containing LIC site and a second can be cloned using the *Bse*RI-containing LIC site.

We tested the LIC cloning at each site using PCR products. Three different gene flanks were cloned using *Bsa*I as a linearisation enzyme, and a total of 45 transformants tested using restriction mapping. We obtained 100% efficiency for the *Bsa*I LIC site. Restriction mapping after cloning into the *Bse*IR LIC site showed that only 56% of 55 colonies tested were the required clones, with the others being parental plasmid DNA.

In order to further improve the efficiency of the procedure and reduce screening for the right-hand LIC site, a *sacB* gene was PCR-amplified and inserted in-between the two *Bse*RI sites of the respective LIC sequence (Fig. 2). This produced pNILRB2, which allows the use of sucrose negative selection for the insertion of one of the gene flanks.

Inserting the first fragment using LIC is independent of restriction sites present in the cloned DNA. However, inserting a second fragment (as we planned for a mutagenesis construct), requires that the second linearisation endonuclease used is not present in the fragment already cloned. While this could often be avoided through planning, we made the plasmids more flexible by inserting two additional LIC sites (one for each flank) into pNILRB2 (Fig. 3). A LIC sequence containing two *Sma*I (CCCGGG) recognition sites for linearisation and a region to generate overhangs (Table 2) was inserted between the two *Bsa*I sites of pNILRB2. To generate a fourth LIC site, two *Swa*I (recognition sequence: ATTTAAAT) sites were inserted between the *Bse*RI sites, flanking *sacB* together with a sequence (Table 2) for T_4_-polymerase-dependent overhang generation.

As a final refinement, we added a screening gene for the insertion of left flanks between the *Bsa*I or *Sma*I LIC sites as well. This might not normally be needed with such efficient cloning, but adds initial reassurance that the cloning has been successful. A *lacZ* α-fragment gene was therefore inserted between the nested *Bsa*I/*Sma*I sites creating pNILRB5 (Fig. 3).

### 3.2 Use of the pNILRB vectors to generate *M. smegmatis* unmarked deletion mutants

**Fig. 4:**
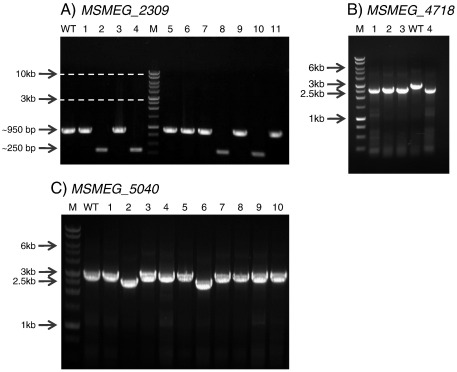
Genomic DNA PCR from isolates of putative deletion mutants. A) for *MSMEG_2309,* B) for *MSMEG_4718* and C) for *MSMEG_5040*. In each case the mutants were identified by a reduction in size of the PCR product (note that the selection of unmarked mutants can result in a wild type or mutant genotype). M: Promega 1 kb ladder, DNA marker; WT: wild type strain; 1–11: potential mutants.

The vectors created were tested by generating suicide delivery constructs with unmarked gene deletions. We produced three mutagenesis constructs for *M. smegmatis* genes (*MSMEG_2309*, *MSMEG_4718* and *MSMEG_5040*, Fig. 4). Mutants were then obtained using a two-step selection process as the previously described for the pNIL series (Parish and Stoker, 2000). In brief, the suicide pNILRB vectors with the appropriate gene flanks and a marker cassette were transformed into *M. smegmatis* by electroporation, and single cross-overs (SCOs) were selected on plates containing hygromycin, kanamycin and X-Gal. Positive colonies (hygromycin^R^/kanamycin^R^/*lac*^+^) were streaked onto antibiotic-free plates to allow for a second cross-over event to occur and double cross-overs (DCOs; sucrose^R^ lac^−^) were isolated through plating on sucrose/X-Gal. The entire cloning procedure could be easily accomplished within 1 week. Mutagenesis in *M. smegmatis* took a further 2 weeks to the point that DCOs were isolated and confirmed by PCR. We have also used the vectors to successfully make mutants with the slow growing mycobacteria.

## 4 Discussion

### 4.1 pNILRB vectors present a robust and rapid cloning strategy

pNILRB5, the final vector of the series, comprises four LIC sites (two available for directional cloning of upstream flanks and two available for directional cloning of downstream flanks), the origin of replication for *E. coli* (*oriE*), a kanamycin resistance gene, a *lacZ* gene for upstream flank insertion selection, a *sacB* gene for downstream flank insertion selection and a unique *Pac*I restriction site for the insertion of the pGOAL19 derived marker cassette for homologous recombination screening. The efficiency of LIC cloning coupled with the presence of the two selection genes in pNILRB5 reduces screening to a minimum, facilitating and speeding the process. We have focused on cloning gene flanks with two steps. In some cases, one-step cloning might be preferred (e.g. the whole gene and flanks are inserted together to mutate single nucleotides), for those situations it is convenient for the backbone vector to have only one selection marker, therefore pNILRB4 (same as pNILRB5 but without the *lacZ* gene) should be used. If gene flanks are to be cloned sequentially then pNILRB5 would be the appropriate choice.

The presence of four LIC sites in the vectors pNILRB4 and pNILRB5 permits 16 different ways of cloning gene flanks, in that there are four pairs of enzymes, two flanks that can be cloned in either site used, and in either order. Furthermore, the *Swa*I site is present only once in the genome of *M. tuberculosis* H37Rv (in the middle of the gene *Rv2023A*). For this reason, these vectors will be usable for inactivating most genes. Consequently, choosing *Bsa*I as the linearisation enzyme for the first LIC site and *Swa*I for the second would be a sensible strategy to avoid enzyme related constraints. Once both flanks are inserted, the last cloning step is the excision of a *Pac*I cassette from pGOAL19 and inclusion into the pNILRB5 *Pac*I site. This is done in the same way as described for the pNIL series(Parish and Stoker, 2000) and it is an efficient process as only one restriction enzyme is used, orientation is irrelevant and *Pac*I sites are rare in mycobacterial genomes (none in *M. tuberculosis*, and two in *M. smegmatis*). The insertion of the *Pac*I cassette adds the equivalent *sacB* and *lacZ* genes to the ones present in pNILRB5, but which will have been removed during the cloning procedure. Although we did not use the *Sma*I and *Swa*I LIC sites for the construction of the delivery vectors mentioned in this study, these sites have been tested and worked efficiently.

The construction of delivery vectors to produce unmarked gene deletions can be a challenging and time-consuming process, and in the past has required a different strategy for each construct, depending on the absence of particular restriction sites in the cloned regions, in order to insert sites at the ends of the amplified region and digest them for cloning. Furthermore, our experience is that the efficiency of digestions with two different enzymes in a multiple cloning site is unreliable, and hard to monitor, and often creates the need for several purification steps during which DNA is lost. The LIC approach used here is both more reliable and easier to troubleshoot, thus allowing a tighter control of the procedure.

In the first place, LIC does not depend on any restriction enzyme digestions of the PCR product. Secondly, the vector can be digested and T_4_-polymerase-treated and stored for use with multiple inserts. Since there are no other problem sources (e.g. overhang compatibility, variable efficiencies of selected enzymes, concentration loss during excessive purification steps, ligation reaction and molecular ratios), this is much more efficient and reproducible than standard cloning.

### 4.2 Ligation-independent cloning appears to be more efficient than standard molecular cloning

The number of recombinant molecules obtained from standard cloning techniques varies greatly depending on several factors, but in particular, the restriction sites used. This restriction sites can influence the procedure in two ways: with the efficiency of the enzyme that recognizes the sequence and with the length and type of overhangs produced.

When cloning flanks with pNILRB (LIC vector without selection markers), we observed 100% frequencies of success for the insertion of upstream flanks (linearisation of the cloning vector with *Bsa*I) and 56% for the insertion of downstream flanks (linearisation of the cloning vector with *Bse*RI). The latter becomes close to 100% when using the negative selection marker, *sacB*. The variation between LIC sites is most likely to be due to the different efficiencies of the linearisation enzymes. We found it harder to achieve complete digestion with *Bse*RI. Gel purification of linear DNA could help to improve efficiencies but we find that unnecessary due to the selection markers we have introduced. This feature is not to do with LIC, which produces very high frequencies of recombination whenever complete linearisation of the vector is obtained (Li and Elledge, 2007). Regardless of the LIC site used we have routinely obtained far higher frequencies of recombinant molecules with LIC than with the standard restriction–ligation approach (in our hands, frequencies obtained varying from 0 to 28.5% using the p1NIL vector).

Generally, it is expected of LIC cloning to be more efficient than standard cloning, since the region of compatibility between overhangs is much longer. LIC overhangs should stick easily together once they establish contact, in contrast, standard molecular cloning relies only on a very small region of complementarity (or no complementarity, for blunt ends) that might not be enough to keep molecules together for long, and relies further on a ligase enzyme that has an associated efficiency and must meet molecules in the stage when they remain annealed.

## 5 Conclusions

We constructed LIC suicide delivery vectors that allow a rapid and efficient cloning strategy in the process of making knock-out mycobacterial strains. pNILRB5 contains four LIC sites and selection/screening to monitor the cloning process. We demonstrated the success of this technique to generate four mutants. We concluded that its use is advantageous over standard molecular cloning by permitting (1) higher cloning efficiencies, (2) fewer steps required, (3) more reproducible and consistent results, (4) a quicker procedure and (5) the use of the technique as a medium throughput method for cloning genes in parallel. We consider that these vectors present a valuable tool for the generation of knock-out mutants through allelic exchange in mycobacteria.

## Figures and Tables

**Table 1:** Bacterial strains and plasmids used in this study.

Strains	Genotype	Source
*Escherichiacoli*		
DH5α	*supE44 ΔlacU169 (ϕlacZΔM15) hsdR17 recA1 endA1 gyrA96 thi-1 relA1*	Invitrogen
*Mycobacterium smegmatis*		
mc^2^155	High frequency transformation mutant ATCC 607	Snapper et al. (1990)
ΔDRKIN	*M. smegmatis* mc^2^155 strain lacking the duplicated chromosomal region	Warner et al. (2006)
Δ2309_Msm_	Δ*2309*_Msm_	This study
Δ4718_Msm_	Δ*4718*_Msm_	This study
Δ5054_Msm_	Δ*5054*_Msm_	This study
Plasmids	Description	Reference

p2NIL	Gene manipulation vector, *Kan*	Parish and Stoker (2000)
pGOAL19	Marker genes, Hyg, ag85p*-lacZ*, hsp60p-*sacB*	This study
pNILRB	p1NIL-derived with 2 LIC sites, *Kan*	This study
pNILRB2	pNILRB-derived with a *sacB* gene to select for downstream gene flanks	This study
pNILRB3	pNILRB2 with an extra LIC site for upstream gene flanks	This study
pNILRB4	pNILRB3-derived with 4 LIC sites, 2 for each flank	This study
pNILRB5	pNILRB4-derived with a *lacZ* gene to select for upstream gene flanks	This study

**Table 2:** Oligonucleotides used for LIC adaptation of p2NIL (LIC sequences are underlined and compatible ends for fragment insertion are in bold).

Primer name	Sequence	Use
*Bse*R1_LIC	GTACTTCCAATCCATAAGCTAGCTTCTCCTC*GGATCC*GAGGAGTTTACTAGTAAGTAAAGGTGGATA**CGTAC**	Insertion of LIC1
*Bse*R1_LIC_COMP	GTATCCACCTTTACTTACTAGTAAACTCCTC*GGATCC*GAGGAGAAGCTAGCTTATGGATTGGAAGTA**CGTAC**	
*Bsa*I_LIC	**ACCGG**TACTTCCAATCCATGGAGACCTCTAGAGGTCTCCAGTAAAGGTGGATAC	Insertion of LIC2
*Bsa*I_LIC_COMP	**ATAGG**TATCCACCTTTACTGGAGACCTCTAGAGGTCTCCATGGATTGGAAGTAC	
*Sma*I_LIC	**CTAGG**TACATCATTTCTCCCGGGTCCTAGGTCCCGGGTATAGATGGTTAC	Insertion of LIC3
*Sma*I_LIC_COMP	**CTAGG**TAACCATCTATACCCGGGACCTAGGACCCGGGAGAAATGATGTAC	
SwaI_LICR	AATAGGAGTACATCCTTTCCATTTAAAT	Insertion of LIC4
SwaI_LICR_COMP	GATATTTAAATGGAAAGGATGTAC	
SwaI_LICL	GGCCATTTAAATTATAGATGGTTAC	Insertion of LIC4
SwaI_LICL_COMP	GGCCGTAACCATCTATAATTTAAAT	

**Table 3:** Oligonucleotides used for PCR amplification in this study (LIC sequences are underlined).

Primer name	Sequence	Use
	*Amplification of gene flanks*	
09U_F1	TACTTCCAATCCATGAGAGAGTCGCCGGTGAACAG	Upstream flank amplification of MSMEG_2309
09U_R1	TATCCACCTTTACTGGACCGCCGGAATGTGATCT	Upstream flank amplification of MSMEG_2309
09D_F1	TATCCACCTTTACTGGCGCCAGTTAGTCACACCA	Downstream flank amplification of MSMEG_2309
09D_R1	TACTTCCAATCCATGCGATCCCGCGATAGTAGGTG	Downstream flank amplification of MSMEG_2309
18U_F1	TACTTCCAATCCATGGCCGTATTCGGCCTTGAA	Upstream flank amplification of MSMEG_4718
18U_R1	TATCCACCTTTACTGCTGCGCCGGTCCGATTT	Upstream flank amplification of MSMEG_4718
18D_F1	TATCCACCTTTACTGAAGGCACAGTCGAGCAC	Downstream flank amplification of MSMEG_4718
18D_R1	TACTTCCAATCCATGTCGACGAGTTCGTGGTCA	Downstream flank amplification of MSMEG_4718
54U_F1	TACTTCCAATCCATGCCACGACTACTTCGTGCTG	Upstream flank amplification of MSMEG_5054
54D_R1	TATCCACCTTTACTGCTTACGCGCGAACAGTGC	Upstream flank amplification of MSMEG_5054
54U_F1	TATCCACCTTTACTGACGATGCTGGCCGATTT	Downstream flank amplification of MSMEG_5054
54D_R1	TACTTCCAATCCATGTACGCGATGGTGTGTGT	Downstream flank amplification of MSMEG_5054
	*Amplification of selective markers*	
*sacB*_F1	GCTGGATCCACCGACGTCCACATATA	Amplification of *sacB* from pNIC28-BSA4
*sacB*_R1	CGTGGATCCAATGCCAATAGGATAT	Amplification of *sacB* from pNIC28-BSA4
*lacZ*_F1	GCTAGCAGCTTGTCTGTAAGCGGATG	Amplification of *lacZ* from pUC18
*lacZ*_R1	GCTAAGCGCCTTTGAGTGAGCTGATACC	Amplification of *lacZ* from pUC18
